# In Response

**DOI:** 10.4269/ajtmh.15-0374b

**Published:** 2015-09-02

**Authors:** Rojelio Mejia

**Affiliations:** Laboratory of Clinical Parasitology and Diagnostics National School of Tropical Medicine Baylor College of Medicine Houston, TX E-mail: rojelio.mejia@bcm.edu

Dear Sir:

We appreciate the response to our article “Intraventricular *Taenia solium* Cysts Presenting with Bruns Syndrome and Indications for Emergent Neurosurgery.” The concern for underreporting the positional symptoms of ventricular cysticercosis as Bruns syndrome is noted, but our search criteria included the term “Bruns” and these other reports did not use that eponym. More importantly, the primary scope of our article was to increase awareness and management for severe cases of intraventricular neurocysticercosis.

Considering enzyme-linked immunosorbent assays (ELISAs), we stated that serological analysis has an adjunct role and that treatment should not be delayed for pending serological studies. Although imaging is the preferred modality for diagnosis, it is not 100% reliable in distinguishing cysticercosis from other neurological masses.^1^ Also, a positive ELISA can provide epidemiologic information for a possible source of infection, as in classic studies from New York City, NY.^2^,^3^

Regarding treatment with anthelmintics before surgery, the data are limited. We did not stress the need for albendazole before surgery, and only one of the two cases received prior treatment. In fact, we stated that medical management should be avoided for patients presenting with neurological emergency. Considering antiparasitic drugs after surgery, it has been reported that small cysts, not removed by surgery, can be missed by magnetic resonance imaging and cause further complications in an already tenuous post-neurosurgical patient.^4^ It has also been reported that postsurgery antiparasitics may help in preventing shunt failures.^5^ Although imaging modalities have greatly improved in resource-rich countries, these may be unavailable in resource-limited areas.

## References

[1] Del Brutto OH, Rajshekhar V, White AC, Tsang VC, Nash TE, Takayanagui OM, Schantz PM, Evans CA, Flisser A, Correa D, Botero D, Allan JC, Sarti E, Gonzalez AE, Gilman RH, García HH (2001). Proposed diagnostic criteria for neurocysticercosis. Neurology.

[2] Schantz PM, Moore AC, Muñoz JL, Hartman BJ, Schaefer JA, Aron AM, Persaud D, Sarti E, Wilson M, Flisser A (1992). Neurocysticercosis in an Orthodox Jewish community in New York City. N Engl J Med.

[3] Moore AC, Lutwick LI, Schantz PM, Pilcher JB, Wilson M, Hightower AW, Chapnick EK, Abter EI, Grossman JR, Fried JA, Ware DA, Haichou X, Hyon SS, Barbour RL, Antar R, Hakim A (1995). Seroprevalence of cysticercosis in an Orthodox Jewish community. Am J Trop Med Hyg.

[4] Mejia R, Nash T (2013). Corticosteroids withdrawal precipitates perilesional edema around calcified *Taenia solium* cysts. Am J Trop Med Hyg.

[5] Gongora-Rivera F, Soto-Hernandez JL, Gonzalez Esquivel D, Cook HJ, Marquez-Caraveo C, Hernandez Davila R, Santos-Zambrano J (2006). Albendazole trial at 15 or 30 mg/kg/day for subarachnoid and intraventricular cysticercosis. Neurology.

